# Diagnostic support for selected neuromuscular diseases using answer-pattern recognition and data mining techniques: a proof of concept multicenter prospective trial

**DOI:** 10.1186/s12911-016-0268-5

**Published:** 2016-03-08

**Authors:** Lorenz Grigull, Werner Lechner, Susanne Petri, Katja Kollewe, Reinhard Dengler, Sandra Mehmecke, Ulrike Schumacher, Thomas Lücke, Christiane Schneider-Gold, Cornelia Köhler, Anne-Katrin Güttsches, Xiaowei Kortum, Frank Klawonn

**Affiliations:** Department of Pediatric Hematology and Oncology, Hannover Medical School, Carl-Neuberg Str. 1, D-30623 Hannover, Germany; Improved Medical Diagnostics, IMD GmbH, Hannover, Germany; Department of Neurology, Hannover Medical School, Hannover, Germany; DRK Clementinenkrankenhaus, Hannover, Germany; Klinik für Kinder- und Jugendmedizin im St. Josef Hospital, Ruhr- Universität Bochum, Bochum, Germany; Department of Neurology, Heimer-Institute at the BG University-Hospital Bergmannsheil GmbH, Ruhr- University Bochum, Bochum, Germany; Ostfalia University of Applied Sciences, Wolfenbuettel, Germany; Helmholtz Centre for Infection Research, Biostatistics Group, Braunschweig, Germany

**Keywords:** Diagnostic support, Rare neuromuscular diseases, Data mining, Questionnaire

## Abstract

**Background:**

Diagnosis of neuromuscular diseases in primary care is often challenging. Rare diseases such as Pompe disease are easily overlooked by the general practitioner. We therefore aimed to develop a diagnostic support tool using patient-oriented questions and combined data mining algorithms recognizing answer patterns in individuals with selected neuromuscular diseases. A multicenter prospective study for the proof of concept was conducted thereafter.

**Methods:**

First, 16 interviews with patients were conducted focusing on their pre-diagnostic observations and experiences. From these interviews, we developed a questionnaire with 46 items. Then, patients with diagnosed neuromuscular diseases as well as patients without such a disease answered the questionnaire to establish a database for data mining. For proof of concept, initially only six diagnoses were chosen (myotonic dystrophy and myotonia (MdMy), Pompe disease (MP), amyotrophic lateral sclerosis (ALS), polyneuropathy (PNP), spinal muscular atrophy (SMA), other neuromuscular diseases, and no neuromuscular disease (NND). A prospective study was performed to validate the automated malleable system, which included six different classification methods combined in a fusion algorithm proposing a final diagnosis. Finally, new diagnoses were incorporated into the system.

**Results:**

In total, questionnaires from 210 individuals were used to train the system. 89.5 % correct diagnoses were achieved during cross-validation. The sensitivity of the system was 93–97 % for individuals with MP, with MdMy and without neuromuscular diseases, but only 69 % in SMA and 81 % in ALS patients. In the prospective trial, 57/64 (89 %) diagnoses were predicted correctly by the computerized system. All questions, or rather all answers, increased the diagnostic accuracy of the system, with the best results reached by the fusion of different classifier methods. Receiver operating curve (ROC) and *p*-value analyses confirmed the results.

**Conclusion:**

A questionnaire-based diagnostic support tool using data mining methods exhibited good results in predicting selected neuromuscular diseases. Due to the variety of neuromuscular diseases, additional studies are required to measure beneficial effects in the clinical setting.

**Electronic supplementary material:**

The online version of this article (doi:10.1186/s12911-016-0268-5) contains supplementary material, which is available to authorized users.

## Background

Patients with late-onset Pompe disease [glycogen storage disease II, acid-maltase deficiency (MP)], motor neuron disease, muscular dystrophy, or other neuromuscular diseases frequently experience diagnostic delay [1–4]. The rarity of these diseases together with clinical variability, atypical presentations, or lack of time for a thorough examination and medical history taking contribute to the delay in diagnosis. In patients with late-onset MP, the diagnostic latency can be more than 20 years [5]. For amyotrophic lateral sclerosis (ALS) patients, the median time from onset of first symptom to diagnosis has been reported to be 11 months [6, 7].

The past medical history offers important clues for diagnosing neuromuscular diseases. Indeed, medical history taking is one of the oldest arts in medicine, but introduction of new reimbursement systems has resulted in less time for communication between physicians and patients and relatives [8]. One goal of this study was therefore to integrate the past medical history into a diagnostic tool and to combine it with modern statistical technologies. In addition, to incorporating the patient’s point of view, we explored the past medical history using questions that were created systematically following interviews focusing on the pre-diagnostic time period. Likewise, the practical experiences of the patient should be closely integrated into the diagnostic process.

We aimed to develop a computerized diagnostic support tool for earlier identification of neuromuscular diseases. In our previous work, we exploited useful scenarios for medical diagnostic support and generated a novel diagnostic support tool for the pediatric emergency department [9]. This ‘emergency tool’ used 14 clinical (e.g. body temperature, blood pressure, pain) and 12 laboratory parameters (e.g. blood count, CRP level, blood-gas analysis) to produce a possible diagnosis. In this study, the system had a diagnostic accuracy between 81 and 97 % for 17 diagnoses such as meningitis, appendicitis, and pneumonia. Although successful, this tool excluded important parts of the past medical history. Therefore, we intended to develop a tool focusing on patients’ perceptions and experiences. In the current project for diagnostic support for individuals with selected neuromuscular diseases; we incorporated patients’ pre-diagnostic experiences and observations to collect answer patterns using questionnaires. Data mining methods then proved to be a reliable tool for answer pattern recognition. This novel tool could serve as diagnostic support for general practitioners (GP) to shorten the diagnostic time in patients with uncommon neuromuscular diseases.

## Methods

### Study design and interviews

In this multicenter prospective pilot study, we tested whether the patient experience explored via a questionnaire could provide diagnostic support for selected rare neuromuscular diseases characterized by long diagnostic latency. First, to gain insight into the patient’s viewpoint during pre-diagnostic phase, interviews with 16 patients with different neuromuscular diseases [MP, ALS, and muscular dystrophy (MD)] were performed across Germany between September 2011 and February 2012 by two authors (US and LG). These semi-structured (narrative) interviews lasted between 45 min and 2.5 h and started with the same initial question (“Please tell us everything that comes to mind before your diagnosis was established. Relay to us everything you consider to be of any importance: your observations and experiences that you would like to share”). At the conclusion of the patient’s narrative, the interviewer could ask additional questions to elucidate more details.

All interviews were digitally recorded, transcribed, and analyzed according to Colaizzi’s techniques [10]. Consequently, an inductive system of categories was developed reflecting the pre-diagnostic phenomena (experiences, symptoms, and/or observations). Examples of pre-diagnostic phenomena are given in Table 1. The process of how the interviews were analyzed to yield a question is illustrated in Supplemental Table 1 for one category.

**Table 1:** Examples for pre-diagnostic experiences and the process of categorization

Patient experience/citation	Category	Question
“My husband enjoys hiking, but for me, steep trails were extremely difficult to manage. I needed to rest often and he would get impatient and cross with me. But what could I do – there was simply no strength in my legs!”	Gait/gait pattern	Can you easily walk uphill?
“Sports in school were simply a nightmare for me. Youth sport meets or any competitive sport exasperated me. Especially those activities that required quick movements were a major fail for me”	Sport activities and training	When you were young were you able to keep up in sports?
“During military service we were forced to pass a fitness course. In addition to other challenges, we had to climb over a six-foot wall. Lifting my body over the barrier was impossible. So I waited until the sergeant was not looking and I would instead run around the barricade.”	Conscious or unconscious compensation of disability	Did you have to “cheat” such as using alternative muscles when performing certain activities?

### Ethical considerations

The ethics committees of Hannover medical university (Ethikkommission der Medizinischen Hochschule Hannover, head: Prof. Dr. H.D. Tröger) and Bochum medical university (Ethik-Kommission der Ruhr Universität Bochum, head: Prof. Dr. M. Zenz) approved the study. All patients gave informed consent for the interviews and all individuals answering the questionnaire gave their informed consent to participate.

### Systematic analysis of the interviews and creation of a questionnaire

Two researchers (US and LG) reviewed and analyzed the interviews. Utilizing techniques described by Colaizzi, patients’ observations were then systematically categorized. A stepwise qualitative analysis was undertaken, including extraction of significant phrases, reduction of the phrases into their essential structures, generation of a question from the essential structure, and validation of questions by the interviewees. To organize the observations and create a questionnaire that would reflect the important experiences, we classified the content of the interviews into different categories. Additionally, we incorporated an additional step, not part of the Colaizzi’s stepwise analysis, and created a question reflecting the pre-diagnostic experiences (Additional file 1). Based on these categories questions were generated resulting in a questionnaire that reflected all categories. Likewise, the questionnaire reflected all the pre-diagnostic phenomena of the interviewees. In close dialogue with patient support groups, the maximum length of the questionnaire was to have no more than two pages and be able to be completed in less than ten minutes. The answers in the questionnaire were scaled from 1 (“absolutely not true”) to 6 (“completely true”). All interviewees as well as patients who were not interviewed evaluated the questions and made suggestions to improve the comprehensibility of the final version of the questionnaire which consisted of 46 questions. Five questions from the questionnaire are shown in Table 2 and the complete questionnaire is provided in the appendix.

**Table 2:** Example of questions used for diagnosing selected neuromuscular diseases

Q	
1	Were you ever diagnosed with an elevated CK level (creatinkinase, a muscle enzyme)?
2	Have your liver parameter/enzymes ever been elevated without apparent reason?
3	Is it particularly challenging to walk uphill?
4	Do you have difficulties standing up from a crouch?
5	Do you often stumble when you walk or do your feet feel “sticky”?
	Do people describe your walk as “funny” or “particular”?

*Q* question

### Collection of answered questionnaires

After formulating of the questionnaire, patients with an established diagnosis (based on standard criteria) of the selected neuromuscular diseases, i.e. muscular dystrophy and myotonia (MdMy) [including patients with Duchenne and Becker muscular dystrophy, oculopharyngeal muscular dystrophy (OPMD), proximal myotonic myopathy (PROMM), facioscapulohumeral MD, limb-girdle-MD, myotonia congenita Thomsen], MP, spinal muscular atrophy (SMA), ALS, polyneuropathy (PNP), and other neuromuscular diseases [including patients with chronic progressive external opthalmoplegia (CPEO)-plus, polymyositis, Ullrich congenital muscular dystrophy, Miyoshi myopathy, Friedreich ataxia, primary lateral sclerosis (PLS), and spinal and bulbar muscular atrophy (SBMA)] were invited (between March 2013 until November 2013) to complete the questionnaire through our neurological outpatient clinic or via local patient group sites. To facilitate participation, a web-based platform was created to answer the questionnaire. Individuals without neuromuscular disease are interpreted as a 7th disease group. During this first period, 210 completed questionnaires were collected and used for cross-validation and later as a training set to predict the correct diagnosis for 64 new patients in a second step.

### Prospective study and extension of the system

The second step, a prospective and multicenter study with different neurological clinics was initiated between October 2013 and October 2014. 64 patients with an established diagnosis of MdMy, MP, SMA, ALS, or PNP completed the questionnaire. The questionnaires were answered and collected in different hospitals in Hannover and Bochum, Germany. Only patients with McA disease were contacted via patient groups.

### Data mining techniques

Finding the right diagnosis based on the answer patterns in the questionnaires can be seen multiclass classification problem. The target attribute was the diagnosis and the elements used for the prediction were the answers to the questions which are given on an ordinal scale. Most classifiers are designed to handle either numerical or categorical attributes. Therefore, the ordinal scale was interpreted as a numerical scale.

Classifiers are based on different assumptions of how the classes – the diagnoses – can be identified or separated. For instance, linear discriminant analysis is based on the assumption that each class is represented by a multivariate normal distribution whereas a decision tree assumes that the classes can be separated by axes-parallel hyper-planes. None of these assumptions really fits the questionnaire data set. Therefore, no single classifier was chosen but rather an ensemble of classifiers.

Classifier ensembles [32] (i.e. combinations of different classification algorithms) often lead to better predictions. The application of classifier ensembles in the context of support for medical diagnosis has been described previously [9]. In the current study, however, we used a combination of eight distinct classifiers (support vector machine, artificial neural network, fuzzy rule-based, random forest, logistic regression, linear discriminant analysis, naive Bayes, and nearest neighbor) to enhance the accuracy of the diagnosis. Selecting the six classifiers is based on the authors’ experience gathered by medical data evaluation for many years.

Although various classifiers are available, there are main groups with a similar underlying mathematical concept. The selected classifiers implement different mathematical assumptions and a diversity of algorithm structures.

In a first step the evaluation of a single questionnaire was performed by six different classifier algorithms. For a patient showing specific symptoms with respect to one of the seven diagnoses, a majority of the 6 classifiers returned an identical result. The classifier results are a vector of probability values for each of the seven diagnoses.

For most questionnaires a fusion algorithm was necessary to perform a weighted majority voting. Each classifier delivered a disease number as well as a corresponding probability value for each assumed diagnosis. The maximum total sum of all probability values for each single diagnosis indicated the diagnosis with the highest relative probability. Summing the probabilities of all classifiers for each diagnosis yielded a score. The diagnosis with the highest score was chosen if it exceeded a certain value.

With the probability p(d,c) for the diagnosis d calculated by the classifier c (c = 1,…6) the diagnosis of the fusion classifier is given by:$\underset{d}{\mathrm{argmax}}\ \left\{{\displaystyle \sum_{c=1}^6}p\left(d,c\right)\right\}$

Evaluation of the classifier ensemble was based on a 21-fold stratified cross-validation algorithm and on case studies with patients who entered the hospital without knowing the final diagnoses. The models were developed and tested by Java software sources including function calls to the R statistics software package libraries.

## Results

### Selection of important pre-diagnostic experiences from the interviews

Many patients experienced a long pre-diagnostic time, especially those with MP and some with MDs. In the interviews, pre-diagnostic experiences were collected and categorized (Table 1). Among various narratives belonging to same category, the questions were created to generate a questionnaire consisting of 46 items reflecting all categories (Table 1 and Additional file 1).

### Creation of a novel questionnaire

The six most important questions in this study are displayed in Table 1 (the complete questionnaire is available as Additional file 2).

### Building a database and the training period

In total, 274 individuals (210 individuals for the training data set, 64 new data sets with a diagnosis of MdMy, ALS, MP, PNP, or SMA) completed the questionnaire. The return rate of the questionnaire differed between the diagnostic groups (Table 2). Most questionnaires were answered through the web-based program between March and May 2013.

During the first study period, 210 answered questionnaires were collected and used for cross-validation. Due to the limited size of the data set, we deviated from the standard 10-fold cross-validation and applied 21-fold cross-validation in order to always have more samples in the training set. The specific number 21 was chosen simply because 21 is a divisor of 210. The 21-fold stratified method selects ten patients for each validation step and repeats this procedure for all 21 groups. Then a classifier was built based on all 210 patients. Later on further 64 new patients filled in the questionnaire and we applied this classifier to these patients, who did not belong to the training data set of the 210 former patients.

### Diagnostic accuracy of the system

#### Results of the training set

For validation purposes, the stratified k-fold cross-validation, a standardized method used in data mining, was used for k = 21. In the group of 210 individuals, 89.5 % (+/- 10.7 %) or 188/210 questionnaires were assigned to the correct diagnosis. The misclassification rate varied between the different classifiers and disease groups (Table 3). The fusion classifiers provided the best results. Here, the diagnostic sensitivity for the detection of MD was 96 and 93 % for MP, but only 69 % for patients with spinal muscular atrophy. The compiled category “other” including a variety of different neurological diseases exhibited the second worst results (81 % correct diagnoses, Table 4).

**Table 3:** Study population (training data set, *n* = 210; prospective data of known diagnoses, *n* = 64)

Diagnostic group	Number of questionnaires (retrospective)	Number of questionnaires (prospective)	Number of questionnaires (total)
Diagnosis 1 (muscular dystrophy/myotonia, MdMy)^a^	50	10	60
Diagnosis 2 (Pompe disease, MP)	43	2	45
Diagnosis 3 (spinal muscular atrophy, SMA)	16	4	20
Diagnosis 4 (amyotrophic lateral sclerosis, ALS)	27	17	44
Diagnosis 5 (polyneuropathy, PNP)	22	23	45
Diagnosis 6 (other neuromuscular diseases, OND)^b^	16	8	24
Diagnosis 7 (no neuromuscular disease, NND)	36	0	36
Total	210	64	274

^a^Including patients with Duchenne and Becker muscular dystrophy (MD), oculopharyngeal muscular dystrophy (OPMD), proximal myotonic myopathy (PROMM), facioscapulohumeral MD, progressive MD, limb-girdle-MD, myotonia congenita Thomsen

^b^Including patients with chronic progressive external opthalmoplegia (CPEO)-plus, polymyositis, Ullrich congenital muscular dystrophy, Miyoshi myopathy, Friedreich ataxia, primary lateral sclerosis (PLS), spinal and bulbar muscular atrophy (SBMA)

**Table 4:** Sensitivity (%) of different classifiers in selected neuromuscular disease groups during 21-fold cross-validation

Diagnostic group Classifier system	MdMy^a^ (1)	MP (2)	SMA (3)	ALS (4)	PNP (5)	Other^b^ (6)	NND^c^ (7)
SVM	92	84	38	78	77	56	89
RF	100	93	69	93	73	63	97
LR	84	86	56	81	73	81	94
NB	94	88	56	52	73	75	92
LD	94	88	69	81	77	81	94
NN	76	81	44	52	64	50	94
Fusion	96	93	69	81	86	81	97

*SVM* support vector machine, *RF* random forest, *LR* logistic regression, *NB* naive Bayes, *LD* linear discriminant analysis, *NN* nearest neighbor

^a^including patients with Duchenne and Becker muscular dystrophy (MD), oculopharyngeal muscular dystrophy (OPMD), proximal myotonic myopathy (PROMM), facioscapulohumeral MD, progressive MD, limb-girdle-MD, myotonia congenita Thomsen

^b^including patients with chronic progressive external opthalmoplegia (CPEO) -plus, polymyositis, Lambert-Eaton myasthenic syndrome, Ullrich congenital muscular dystrophy, Miyoshi myopathy, Friedreich ataxia, primary lateral sclerosis (PLS), spinal and bulbar muscular atrophy (SBMA), and chronic inflammatory demyelinating polyneuropathy (CIDP)

^c^ *NND* no neuromuscular disease

In Table 5, a confusion matrix is shown for the results of the fusion classifier which combines the results of 6 data mining methods. Depending on the number of patients and the type of disease in each group the positive predictive value (PPV) and the negative predictive value (NPV) vary between 0.83 and 1 for the PPV and between 0.97 and 0.99 for the NPV.

**Table 5:** The fusion classifier exhibits good PPV and NPV based on 21-fold cross-validation

	MdMy (*n* = 50)	MP (*n* = 43)	SMA (*n* = 16)	ALS (*n* = 27)	PNP (*n* = 22)	other (*n* = 16)	NND (*n* = 36)
	48	3	3	1	0	1	0
	2	40	0	0	0	0	0
	0	0	11	0	1	1	0
	0	0	0	22	2	0	0
	0	0	1	1	19	1	1
	0	0	0	0	0	13	0
	0	0	1	3	0	0	35
PPV	0.87	0.95	0.85	0.92	0.83	1	0.90
NPV	0.98	0.98	0.97	0.97	0.98	0.98	0.99

To illustrate the variety between different classifying systems, the results of different data mining methods during cross-validation was calculated. The results of the final fusion classifier were better than the results of single classifiers in most of the layers, indicating that the combination of different classifiers outperforms any single classifier in this setting.

The amount of questions gathered by the Colaizzi method guarantees high sensitivity values reached by the data mining algorithms. In test evaluations a stepwise reduction of the number questions was investigated with the result that the sensitivity rates decline with the number of omitted questions. However, the rate of the decline depends on the impact of a single question on the sensitivity values measured by ROC/AUC (area under the curve) values and *p*-value computations.

Question 40 (“Is it true that physical activities that you used to be able to do are not possible anymore?”) serves as an example for a “weaker” question. For diagnosis 1, question 40 shows a weak *p*-value for the corresponding coefficient in logistic regression. If this question is skipped, the sensitivity value for this diagnosis declines from 96 % down to 92 %, while the averaged sensitivity value for all diagnoses performs with only 1 % decrease. Cancelling “weak” questions with less significant *p*-values leads to a moderate decrease in the total sensitivity values, but triggers strong reduction of sensitivity for single diagnoses. Therefore, the evaluations are based on the full amount of 46 questions collected by the Colaizzi method.

The *p*-values for the questions and the seven diagnoses under investigation are shown in the Additional file 3.

#### Results of the prospective trial

During the one-year prospective trial, 64 patients with a diagnosis of MdMy, MP, SMA, ALS, or PNP answered the questionnaire (Table 6). In this group, 89 % correct diagnoses were provided. The distribution in the different disease groups varied. Especially in patients with PNP, there was a relevant rate of incorrect diagnoses.

**Table 6:** Diagnostic results during the prospective trial in 64 patients

	MdMy	MP	SMA	ALS	PNP	other	no
MdMy	10	1	0	0	1	1	0
MP	0	1	0	0	0	0	0
SMA	0	0	4	0	2	0	0
ALS	0	0	0	17	0	0	0
PNP	0	0	0	0	18	0	0
other	0	0	0	0	2	7	0
no	0	0	0	0	0	0	0
total	10	2	4	17	23	8	0

*MdMy* muscular dystrophy, *MP* Pompe Disease, *SMA* spinal muscular atrophy, *ALS* amyotrophic lateral sclerosis, *PNP* polyneuropathy; other see Table 2

#### Results in patients without a diagnosis at first encounter

Nine individuals were included into the trial, who did not have a definitive diagnosis at the time of completion of the questionnaire. They were referred to a tertiary center to establish a final diagnosis based on symptomology. There was suspicion for a neuromuscular disease by the referring neurologist, but the diagnosis needed confirmation at a tertiary clinic. Two out of four patients, later confirmed to have PNP, received the correct diagnosis employing the computer program. The remaining two PNP patients were incorrectly classified as SMA. One patient subsequently diagnosed with ALS, was correctly diagnosed by our system. Four patients with diagnoses unknown to the system (vasculitis, MG, rhabdomyolysis, and polymyositis) were correctly classified as “other neuromuscular diseases”.

### Results of the ROC curves, AUC values, confusion matrix and *p*-values

Receiver operating characteristic (ROC) curves and the area under the ROC curve (AUC) were also used to evaluate the predictive power of our approach. Finally, a validation of the importance of the questions was carried out. On the one hand, a *p*-value for each question was calculated based on the significance (deviation from zero) of the corresponding coefficient in logistic regression (Additional file 3). On the other hand, the performance of the system was tested by leaving out questions with less significant *p*-values.

**Fig. 1:**
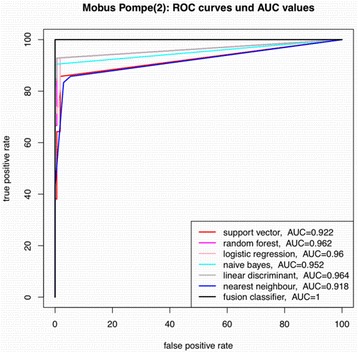
ROC curves and AUC values indicate variable diagnostic sensitivity among different classifier systems for identifying patients with Pompe disease. The results are based on the training set of 210 questionnaires during cross validation. The best classifier results were obtained with the fusion classifier (black line, 100 % correct diagnoses), which identified all 43 Pompe patients during the 21-fold stratified cross-validation runs

Figure 1 illustrates the high diagnostic accuracy of different classifiers with the best results for the fusion classifier for individuals with Pompe disease.

## Discussion

The main findings of this study are that patients with selected neuromuscular diseases could be identified or distinguished using data mining in conjunction with answer pattern analysis from newly developed questionnaire. Secondly, the results of the study support the notion that data mining methods show plasticity and expandability, making this approach a promising tool for modern diagnostics. Indeed, the diagnostic accuracy of the tool was nearly 90 % depending on the diagnostic group. Good results for NPV and PPV could be reached but need confirmation in a larger scale study. These preliminary results support our hypothesis that medical history taking, which was simulated here using selected questions, together with modern computational methods is powerful to assist the physician in generating a diagnosis.

Diagnostic support is needed for neuromuscular diseases due to a lack of experience with these disease entities by GPs and even many sub-specialties. Often the diagnosis is delayed. A recent report on patients with oculopharyngeal muscular dystrophy by Scotland et al. demonstrated a prolonged time frame, up to 20 years, before the diagnosis was made [2]. The reasons for the delay were multiple including patient denial, nonspecific symptoms, clinical variability, and rarity of the disease [13–16]. However, the role of the GP as gatekeeper must be highlighted as well [1, 11, 12]. New systems to remind medical gatekeepers of rare diseases are highly desirable and multiple reports addressing delays in diagnosis in different disease groups underscored this issue [6, 7, 11].

Computer aided diagnostic support dates back to the 1980s [17]. Using databases and statistical algorithms, scientists attempted to reduce diagnostic mistakes and enhance diagnostic accuracy [18–21]. Despite some success, daily real life application was limited and most diagnoses are still made by the practitioner without the assistance of computerized programs. In addition, these initial computer based diagnostic tools had drawbacks. First, the programming of rules to update any expert system is time-consuming and the number of rules to be incorporated in such a system rises exponentially such that data entry is often impracticable [22]. Moreover, self-assessment by doctors has the potential to inadvertently reinforce false concepts to the detriment of excluding other plausible ideas [23–27]. These barriers were successfully addressed in our project by utilizing self-learning data mining methods and transferring the data entry to patients who simply answer the questionnaire while waiting to see the doctor. This structure also takes advantage of the patient as being an expert on his/her own health.

Unfortunately, the clues for diagnosis are often lost in the physician-patient communication or the physician simply do not appreciate the patient’s perspective fully [28, 29]. Exploring the past medical history thoroughly is a cornerstone of the medical evaluation, but it is hampered by lack of time and misunderstanding between health professionals and patients [30, 31]. On the other hand, patients with rare chronic diseases are experts in detecting the signs and symptoms of their disease. Careful attention to patients’ experiences as related to their disease gives important hints for additional work up. These ideas were successfully integrated into our diagnostic support tool using questions developed from patients’ pre-diagnostic experiences [29].

The diagnostic delay in patients with neuromuscular disorders is influenced by the treating physician at first encounter [2]. A neurologist might not need a diagnostic support tool for detecting neuromuscular diseases, but for a GP this could be different. The patient with certain key symptoms (e.g. fatigue, cramps, muscle twitching/fasciculations, tripping, slurred speech, or muscle weakness) could answer the questionnaire in the waiting room. The putative diagnosis would be immediately displayed to the physician who could then consider the suggested diagnosis and explore the past medical history in more detail to help refute or substantiate the diagnosis and request additional laboratory or radiological exams prior to referring the patient to a subspecialist.

Our study has certain limitations, however. First, we conducted interviews and collected questionnaires on a heterogeneous group of individuals and the number was small. This might have resulted in a selection bias of the final questions. Importantly, some observations are not reflected in the current questionnaire. Although this may reflect the daily work of a GP who cannot ask all possible questions, it also reveals the restraints of a questionnaire-based diagnostic tool. Second, the tool under investigation does not render a definitive diagnosis but rather directs the GP to a diagnostic group. The treating physician can prompt further testing to reach a definite diagnosis. Of note, we choose only six neuromuscular diseases where diagnostic delay is common, but many other conditions with similar symptoms cannot be diagnosed with this tool at the current time. In addition, one might criticize the system for overfitting and as such being biased for detecting certain diseases much better than detecting a simple muscle ache. However, this may be partially remedied by prospective testing and expansion of the system with new diagnoses (e.g. McA, MMN, and IBM). However, the pilot evaluation of nine patients without a diagnosis resulted in high quality diagnostic suggestions. Third, the prospective trial included only patients with an established neuromuscular disease but no other diagnoses, e.g. chronic cardiac or pulmonary diseases, mimicking a neuromuscular disorder.

The training data set of 210 questions as well as the prospective tests with 64 patients was relatively small and did not represent all possible disease manifestations or all possible neuromuscular diseases. Particularly in the group of patients with muscular dystrophies, we collected questionnaires from patients with different diagnoses who were then computed into one larger group, resulting in more heterogeneity in the group. The next challenge for the system will be to detect individuals with fibromyalgia and pulmonological or psychosomatic disorders, which will be addressed in a future trial. However, as a surprising proof of concept, our data showed that it is possible to generate a diagnostic hint of neuromuscular diseases by computer-based analysis of answer patterns. In contrast, internet search engines of symptoms for self-diagnosis showed disappointing results for motor neuron diseases [33]. The application of data mining techniques improved the diagnostic quality in selected clinical scenarios [34]. Recently, the combination of questionnaires and data mining techniques proved very successful for diagnosing rare pulmonary diseases in children [35]. A randomized study performed by Kostopoulou and co-workers recently demonstrated the beneficial effects of computerized support on the diagnostic accuracy of GPs indicating the potential value of CDSS for clinical usage [36]. A similar study is planned with the tool under investigation here to analyze its benefit for the clinical use.

## Conclusions

In conclusion, these preliminary data indicate that individuals with selected neuromuscular diseases share symptoms and experiences in the pre-diagnostic period which were exploited to develop a specific questionnaire and subsequent data mining techniques of answer patterns. A diagnostic support tool may help the GP to identify patients with unspecific symptoms that might be the first indication of a rare neuromuscular disease. Today this tool covers only limited diseases and diagnostic categories. Therefor this system is not yet ready for the clinical use. Further trials are needed before this system may be integrated into routine clinical use.

## Additional files

Additional file 1: Test data set of the study. (DOCX 16.2 kb) Additional file 2: Training data set of the study. (DOC 26.5 kb) Additional file 3:
*P*-value calculations for questions in different disease groups. (TIF 1.80 mb)

## Abbreviations

(CPEO)-plus: chronic progressive external opthalmoplegia
ALS: amyotrophic lateral sclerosis
AUC: area under the curve
CIDP: chronic inflammatory demyelinating polyneuropathy
GP: general practitioner
IBM: inclusion body myositis
MD: muscular dystrophy
MdMy: myotonic dystrophy and myotonia
MG: myasthenia gravis
MMN: multifocal motor neuropathy
MP: Pompe disease
NND: no neuromuscular disease
OND: other neuromuscular diseases
OPMD: oculopharyngeal muscular dystrophy
PLS: primary lateral sclerosis
PNP: polyneuropathy
PROMM: proximal myotonic myopathy
ROC: receiver operating curve
SBMA: spinal and bulbar muscular atrophy
SMA: spinal muscular atrophy
