# Supplementary material for: Diagnostic support for selected neuromuscular diseases using answer-pattern recognition and data mining techniques: a proof of concept multicenter prospective trial
Source: BMC Med Inform Decis Mak. 2016 Mar 8;16:31. doi: 10.1186/s12911-016-0268-5 (PMC4782522; doi:10.1186/s12911-016-0268-5)
Supplement: Additional file 1: — Test data set of the study. (DOCX 16.2 kb) [file 12911_2016_268_MOESM1_ESM.docx]

Supplemental file 1. Examples for the analysis according to Colaizzi in the category: ‘Peculiarities and abnormalities in sport activities’

| Step 1  quotation | Step 2  meaning | Step 3  category | Step 4  essential structure and exhaustive description of the phenomenon | Step 5  question |
| --- | --- | --- | --- | --- |
| ‚Since I‘m young, I never was a sporting ace. Well, I did sport and I tried to keep up with my mates, but my performance was always inferior compared to friends or classmates and I was never really good in sport activities‘. | In sport activities the level of performance of mates were never achieved | Peculiarities and und abnormalities in sport activities | Despite regular sport activities no success is achieved | When you were young were you able to keep up at sports? |
| ‘There were times when I went four times per week for training, but there was no improvement, really no improvement at all!’ | I did a lot of exercise without amelioration of the performance | Peculiarities and und abnormalities in sport activities | Improvement of performance is lacking despite training | Can you improve your performance by training activities? |
| ‘I remember since I was young that mountain climbing or hiking and walking a steep path – I never could do that (…)’ . | It was noted since childhood that walking in hilly area is very difficult | Peculiarities and und abnormalities in sport activities | Walking in hilly area is difficult or impossible | Does it feel extraordinarily hard to walk uphill? |
| ‚I always tried very hard but it simply didn’t work out. And then there were these terrible sportsmeeting at school – running, jumping, throwing – I tried very, very hard to succeed, but every sport that had to do with spontaneous energy like sprint or broad jump – nil return, nothing, no success – it was dreadful for me.’ | I always tried hard, but couldn’t fulfill the requirements in school sport activities.  All sport activities that require bursting of energy could not be mastered | Peculiarities and und abnormalities in sport activities | Despite all efforts no success   No success in sport activities that require spontaneous bursting of power | Do certain activities that require a quick onset of movement (like sprints, throwing, jumping) seem harder?  Do you feel like ‘you want but you can’t’ when it comes to exercise? |
