# Supplementary material for: Diagnostic support for selected neuromuscular diseases using answer-pattern recognition and data mining techniques: a proof of concept multicenter prospective trial
Source: BMC Med Inform Decis Mak. 2016 Mar 8;16:31. doi: 10.1186/s12911-016-0268-5 (PMC4782522; doi:10.1186/s12911-016-0268-5)
Supplement: Additional file 2: — Training data set of the study. (DOC 26.5 kb) [file 12911_2016_268_MOESM2_ESM.doc]

Questionnaire for diagnostic support in selected neuromuscular diseases.

1. Can you easily lift a heavy object off the floor (e.g. case of bottles)?

2. Compared to the past do you feel more exhausted or tired?

3. Can you open a skrew top bottle without difficulties?

4. Has a doctor ever told you your liver parameter/enzymes are elevated?

5. Do often stumble when you walk or do you feel like your feet are „sticky“?

6. Can you lift both legs off the floor when lying flat?

7. Have you got pain after physical work out?

8. Would you have difficulties to jump over a little puddle?

9. Can you put your luggage into over head compartments without difficulties?

10. Do you ever feel your muscles twitching?

11. Do others describe you as lazy/a couch potatoe?

12 If you lose your balance can you catch yourself in time?

13. Is it so that after strenuous days you feel like your muscle strength is dwindling?

14 Are you in constant back ache?

15. Do you have „overbendy“ joints?

16. Were you ever diagnosed with an elevated CK (creatinkinase, a muscle enzyme)?

17. Can you bend your leg to the side?

18. Do you feel like „you want but you can’t“ when it comes to exercise?

19. Do you need a long time to recover from illnesses?

20. When you roll over in bed is your movement different to that of other people?

21 Can you lift your head when lying down without difficulties?

22. Does it feel extraordinarily hard to walk uphill?

23. Do you prefer chairs with armrests because you use them to push yourself up?

24. Have you ever fallen without a reason?

25. Can you stand up from a crouch easily?

26. Do you „throw your legs outwards” whilst walking?

27. Is it hard for you to work overhead?

28. When you were young were you able to keep up at sports?

29. Do you have difficulties with speaking clearly? (lisp, mumbling)

30. Have you noticed a reduced strength in arms and legs?

31. Do you need to use your hand for support when crossing your legs?

32. Do you need momentum to pick up heavy objects off the floor?

33. Do you have difficulties to button up your shirt?

34. Can you jump up from standing?

35. Does it seem to you that your limbs are shrinking/getting smaller?

36. Would small bumps on the floor cause difficulties when walking?

37. Do certain activities that require a quick onset of movement (like sprints, throwing, jumping) seem harder?

38. Do people describe your walk as „funny“ or „particular“?

39. When putting your legs up do you need your arms for support?

40. Is it true that activities that you used to be able to do are not possible anymore?

41. Could you hold a full water bottle on a stretched arm?

42. Do you suffer from cramps?

43. Is it hard for you to carry heavy things, like shopping bags etc.)

44. Have you noticed that your shoulder blades are sticking out („Angel wings“)?

45 Do you have to „cheat“ when doing certain movements by using alternative muscles whilst avoiding others?

46. Have you noticed an increasing abdominal circumference without gaining weight?
