# Supplementary figures and images for: Diagnostic support for selected neuromuscular diseases using answer-pattern recognition and data mining techniques: a proof of concept multicenter prospective trial
Source: BMC Med Inform Decis Mak. 2016 Mar 8;16:31. doi: 10.1186/s12911-016-0268-5 (PMC4782522; doi:10.1186/s12911-016-0268-5)

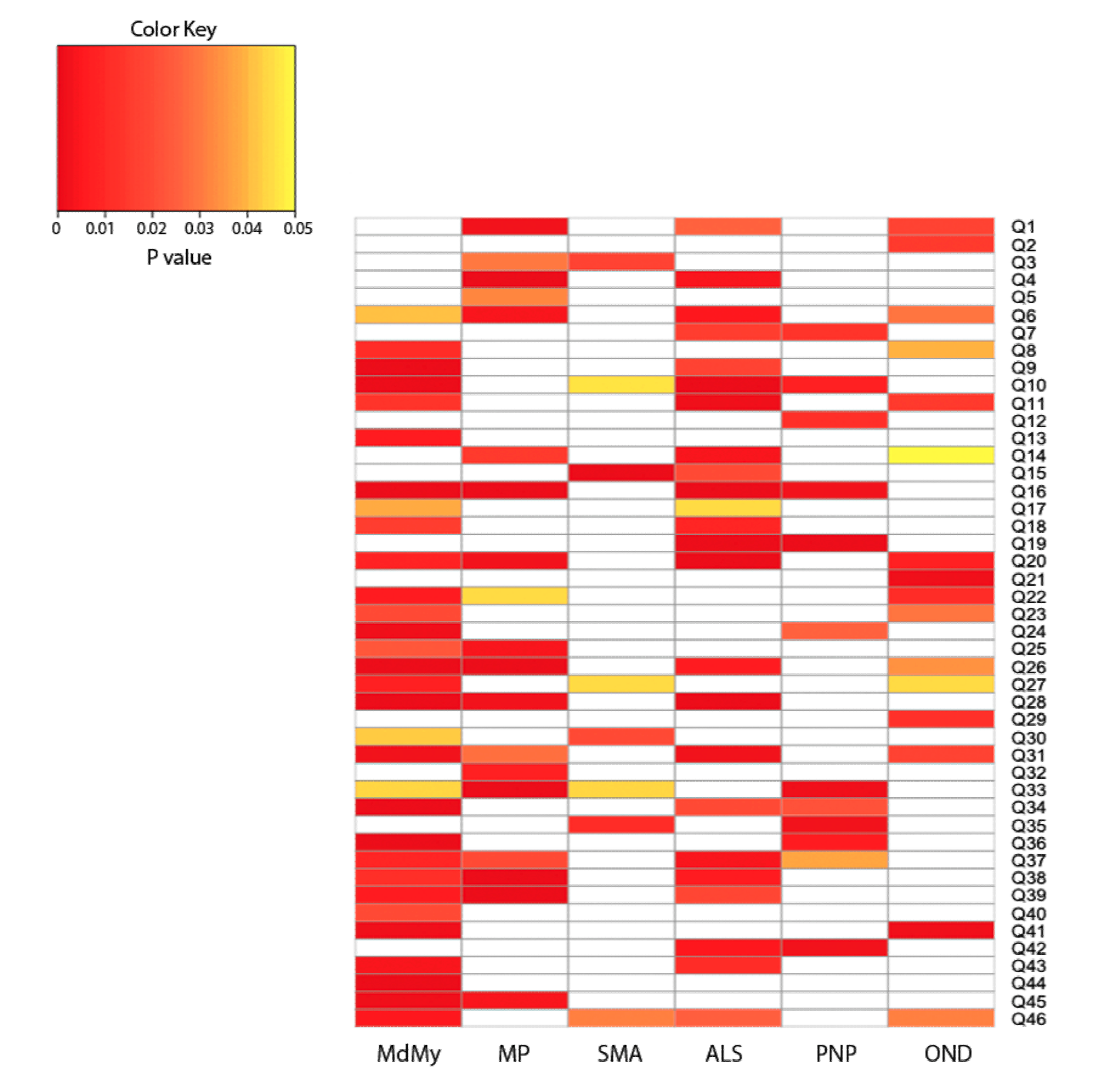

Supplement: Additional file 3: — P-value calculations for questions in different disease groups. (TIF 1.80 mb) [file 12911_2016_268_MOESM3_ESM.tif]
